# Three-Dimensional Analysis of Mitochondrial Crista Ultrastructure in a Patient with Leigh Syndrome by *In Situ* Cryoelectron Tomography

**DOI:** 10.1016/j.isci.2018.07.014

**Published:** 2018-07-20

**Authors:** Stephanie E. Siegmund, Robert Grassucci, Stephen D. Carter, Emanuele Barca, Zachary J. Farino, Martí Juanola-Falgarona, Peijun Zhang, Kurenai Tanji, Michio Hirano, Eric A. Schon, Joachim Frank, Zachary Freyberg

**Affiliations:** 1 Department of Cellular, Molecular and Biophysical Studies, Columbia University Medical Center, New York, NY 10032, USA; 2 Department of Biochemistry and Molecular Biophysics, Columbia University Medical Center, New York, NY 10032, USA; 3 Division of Biology and Biological Engineering, California Institute of Technology, Pasadena, CA 91125, USA; 4 Department of Neurology, Columbia University Medical Center, New York, NY 10032, USA; 5 Department of Psychiatry, University of Pittsburgh, Pittsburgh, PA 15213, USA; 6 Department of Structural Biology, University of Pittsburgh, Pittsburgh, PA 15213, USA; 7 Department of Pathology and Cell Biology, Columbia University Medical Center, New York, NY 10032, USA; 8 Department of Genetics and Development, Columbia University Medical Center, New York, NY 10032, USA; 9 Department of Biological Sciences, Columbia University, New York, NY 10032, USA; 10 Department of Cell Biology, University of Pittsburgh, Pittsburgh, PA 15213, USA

**Keywords:** Organizational Aspects of Cell Biology, Structural Biology, Resolution Techniques

## Abstract

Mitochondrial diseases produce profound neurological dysfunction via mutations affecting mitochondrial energy production, including the relatively common Leigh syndrome (LS). We recently described an LS case caused by a pathogenic mutation in *USMG5*, encoding a small supernumerary subunit of mitochondrial ATP synthase. This protein is integral for ATP synthase dimerization, and patient fibroblasts revealed an almost total loss of ATP synthase dimers. Here, we utilize *in situ* cryoelectron tomography (cryo-ET) in a clinical case-control study of mitochondrial disease to directly study mitochondria within cultured fibroblasts from a patient with LS and a healthy human control subject. Through tomographic analysis of patient and control mitochondria, we find that loss of ATP synthase dimerization due to the pathogenic mutation causes profound disturbances of mitochondrial crista ultrastructure. Overall, this work supports the crucial role of ATP synthase in regulating crista architecture in the context of human disease.

## Highlights

• We used cryo-ET in a case-control study of a patient with LS and healthy mitochondria
• Loss of ATP synthase dimers disrupts crista architecture in LS mitochondria
• Mitochondrial ultrastructure plays a key role in bioenergetic output *in vivo*

## Graphical Abstract

**Figure:**
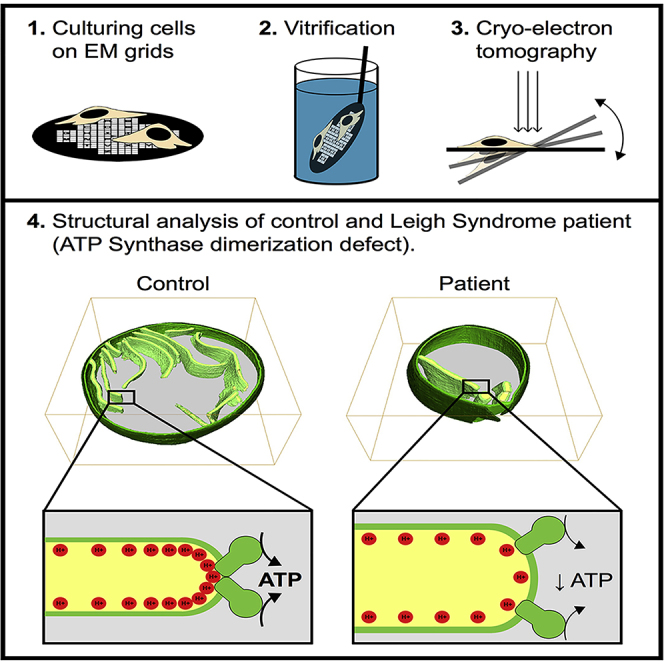

## Introduction

Mitochondrial diseases comprise a group of devastating neurological disorders caused by mutations affecting mitochondrial energy production via oxidative phosphorylation (OxPhos). One of the most common OxPhos disorders is a neonatal- or pediatric-onset subacute necrotizing encephalomyelopathy known as Leigh syndrome (LS) (Lake et al., 2015), which presents with progressive impairment in cognitive and motor functions and premature mortality (Gerards et al., 2016). Of the over 75 unique pathogenic mutations known to cause LS, most encode components of ATP generation (i.e., OxPhos) machinery, including the four mitochondrial respiratory complexes, the ATP synthase, the electron carrier coenzyme Q_10_, and the pyruvate dehydrogenase complex (Lake et al., 2016). More recently, LS and Leigh-like syndrome have been linked to pathogenic mutations in genes encoding proteins regulating crista architecture, including *SLC25A46* (Janer et al., 2016) and *OPA1* (Finsterer and Zarrouk-Mahjoub, 2017). Indeed, LS-causing mutations in *SLC25A46* result in abnormal mitochondrial architecture, including significantly shortened cristae (Janer et al., 2016), whereas disease mutations that reduce *OPA1* expression produce decreased mitochondrial volume density and diminished cristae membrane surface area (Zhao et al., 2017).

We have recently described a case of relatively mild LS in a 7-year-old patient (henceforth “patient”), caused by a novel pathogenic mutation in the gene *USMG5* (*upregulated during skeletal muscle growth protein 5*) that encodes a small ∼6.5-kDa protein termed diabetes-associated protein in insulin-sensitive tissues (DAPIT) (Barca et al., 2018). The mutation abolishes the canonical GT splice site donor of exon 4 and generates aberrant transcripts that are degraded via nonsense-mediated decay. Homozygosity results in <10% of normal transcripts that persist due to leaky transcription (Barca et al., 2018). DAPIT is associated with the mitochondrial respiratory complex V (ATP synthase) (Ohsakaya et al., 2011) as a supernumerary rather than a structural subunit (Chen et al., 2007). Unlike other OxPhos subunits linked to LS, including those within the ATP synthase, this protein has no known role in ATP production per se. Rather, it is thought to localize to the transmembrane domain associated with the peripheral stalk that is required for ATP synthase dimerization (Dudkina et al., 2010, Walker, 2013). Biochemical studies of the patient's fibroblasts revealed (1) no detectable ATP synthase dimers, (2) normal amounts of ATP synthase monomers, and (3) decreased ATP synthase activity (Barca et al., 2018). Indeed, previously reported complete knockouts of the DAPIT protein exhibited ∼40% decrease in ATP synthase enzymatic activity (Ohsakaya et al., 2011). ATP synthase dimers are thought to play an important role in generating the curvature at the apex of mitochondrial cristae (Cogliati et al., 2016). Consistent with this, mutations that disrupt ATP synthase dimerization in yeast cause crista ultrastructural defects in purified yeast mitochondria (Davies et al., 2012). Nevertheless, the precise relationship between crista ultrastructure and ATP synthase dimerization defects in humans remains poorly understood.

Abnormal crista ultrastructure has been correlated with dysfunctions in respiratory capacity, both *in vitro* (Nielsen et al., 2017) and *in vivo* in human skeletal muscle (Hackenbrock, 1966), and has been further linked to human pathology (Acehan et al., 2007, Cogliati et al., 2016; Quintana-Cabrera et al., 2018). Disturbances in mitochondrial ultrastructure have also been characterized in patients with LS using conventional transmission electron microscopy (TEM) of biopsies (generally muscle) (Willems et al., 1977). Although the resolution of TEM data is sufficient to distinguish suborganellar components including the mitochondrial outer membrane (MOM), mitochondrial inner membrane (MIM), and cristae, the relatively harsh sample preparation can distort membranes and cause material loss (Mannella et al., 2013, Winey et al., 2014, Wisse et al., 2010), potentially obscuring the interpretation of genuine biological phenotypes. The recent revolution in cryo-electron microscopy (cryo-EM) has enabled the direct visualization of near-native samples without additional sample manipulations such as chemical fixation (Pilhofer et al., 2010, Thompson et al., 2016). Further combination of cryo-EM with tomographic imaging schemes in cryo-electron tomography (cryo-ET) enables three-dimensional visualization of these specimens (McIntosh et al., 2005, Zick et al., 2009).

With few exceptions, to date, most high-resolution information on mitochondrial structure has been obtained from cryo-ET analyses of purified organelles (Davies et al., 2012, Dudkina et al., 2010, Englmeier et al., 2017), leaving many questions open concerning the effects of disease on mitochondria within the cellular context. While the thickness of biological samples has been a traditional limitation of cryo-EM or cryo-ET imaging, the relatively thin morphology of primary human skin fibroblasts, particularly in the periphery, provides an ideal intact biological environment to image cellular structures *in situ*. Moreover, mitochondria are spheroid or tubular/elongated structures ∼0.2–5 μm in diameter (McCarron et al., 2013, Perkins and Frey, 2000) and are therefore amenable to imaging by cryo-EM and cryo-ET without sectioning, provided that their surrounding environment is similarly thin (Davies and Daum, 2013).

Here, we introduce the application of *in situ* cryo-ET to a clinical case-control study of a human mitochondrial disease. By utilizing cryo-ET to directly study mitochondria within cultured primary fibroblasts from a patient with LS and a healthy human control subject, we found that a pathogenic mutation in DAPIT that ablates ATP synthase dimerization causes profound disturbances of mitochondrial crista ultrastructure, resulting in human disease.

## Results

### Cryo-ET Reveals Abnormal Architecture of Mitochondrial Cristae in Patient Cells

**Figure 1:**
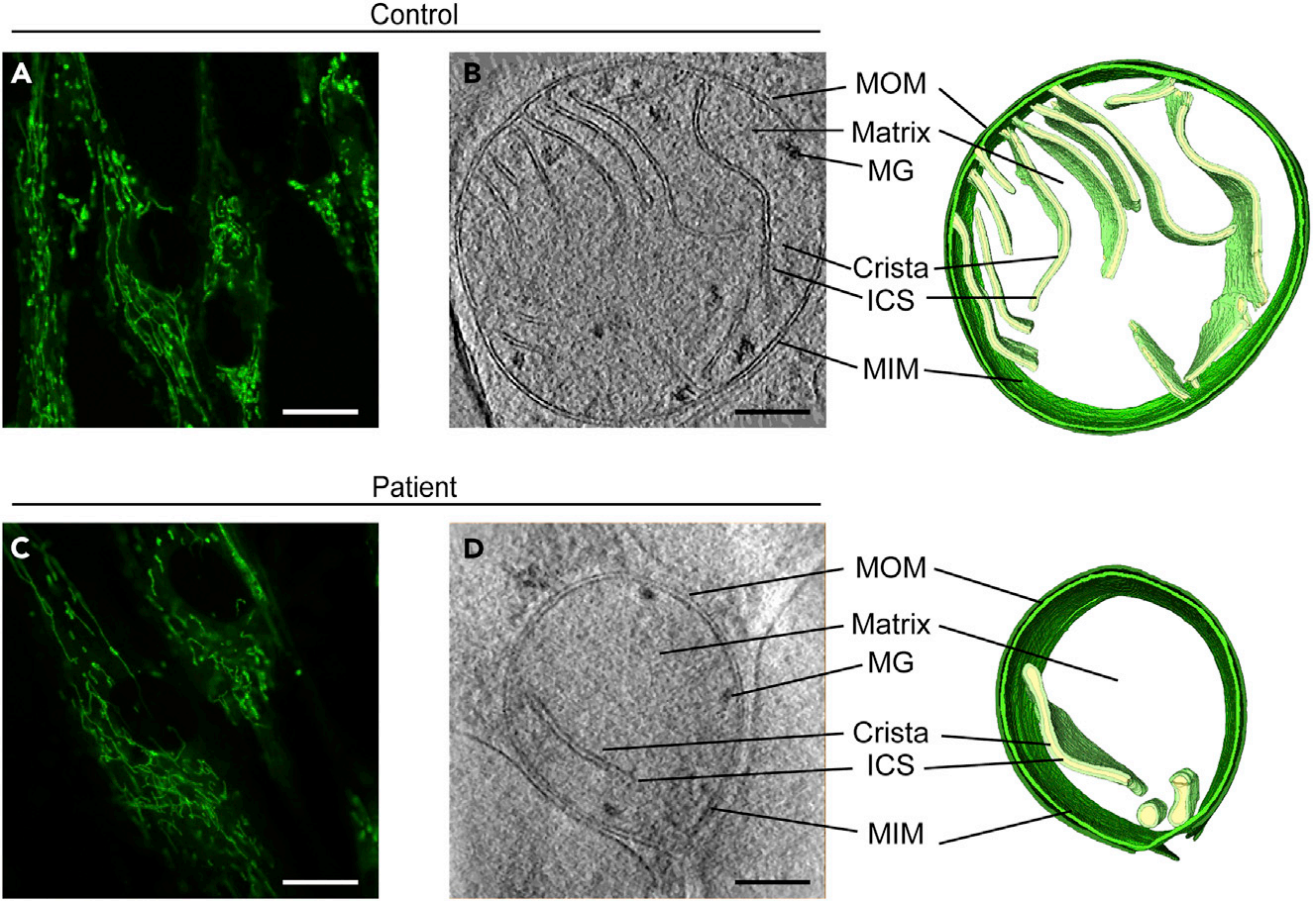
Analysis of Patient Mitochondria Reveals Abnormal Crista Membrane Architecture, Related to Figures S1 and S2 (A and C) Representative fibroblasts from control and patient were stained with MitoTracker Green FM and imaged by live confocal fluorescence microscopy at a magnification of 63×, showing typical morphology of mitochondria. Scale bars represent 5 μm. (B and D) Control and patient mitochondria were imaged by *in situ* cryo-electron tomography at a nominal magnification of 15,500×, and three-dimensional models of each organelle were generated by manual segmentation. Key mitochondrial structural features are indicated, including: mitochondrial outer membrane (MOM), intracristal space (ICS), mitochondrial inner membrane (MIM), and matrix granules (MG), with three-dimensional reconstructions at right. Scale bars represent 200 nm.

We first examined primary skin fibroblasts from a patient with LS and a healthy human control subject by live confocal fluorescence light microscopy. We used the live mitochondrial marker MitoTracker Green FM and saw no obvious differences in mitochondrial shape or distribution (Figures 1A and 1C).

Patient and healthy control fibroblasts were next grown on gold-carbon electron microscopy grids, vitrified, and imaged within sufficiently thin regions of the cell (Figure S1). Tilt series were obtained from patient and control mitochondria (n = 10 for each cell type) from at least three different biological replicates (i.e., independently plunge-frozen cells). Visual inspection of the mitochondria within the individual micrographs from patient and control cells revealed both spherical and tubular/elongated mitochondria, with closely apposed MIM and MOM, and an electron-dense matrix containing cristae and calcium/phosphorus ion granules (Wolf et al., 2017) (Figure S2). The appearance of these mitochondria was comparable to previous reports of *in situ* human mitochondria (Hu, 2014) and consistent across both our patient and control samples. In addition, we found that the signal-to-noise ratio is considerably lower for *in situ* datasets compared with prior work using purified mitochondria (Nicastro et al., 2000), as reflected in such parameters as mitochondrial matrix texture. We therefore also analyzed isolated mitochondria from control and patient fibroblasts. However, since we observed changes in the crista organization compared with our *in situ* samples (data not shown), we decided to focus our analyses on *in situ* data to maximize its biological relevance.

We next reconstructed each tilt series from patient and control mitochondria into three-dimensional volumes (i.e., tomograms) with each tomogram representing an intact mitochondrion within its native environment (Figure S2, Videos S1, S2, S3, and S4). Of note, due to technical limitations inherent to cryo-ET, namely the restriction of tilt series collection to ±70°, objects such as membranes perpendicular to the beam may be poorly resolved (Leis, 2018). Thus, structures such as mitochondria can appear elliptically distorted at their upper and lower margins. We therefore restricted our reconstructions to the middle regions of each tomogram to ensure the accuracy of our analyses.

**Figure 2:**
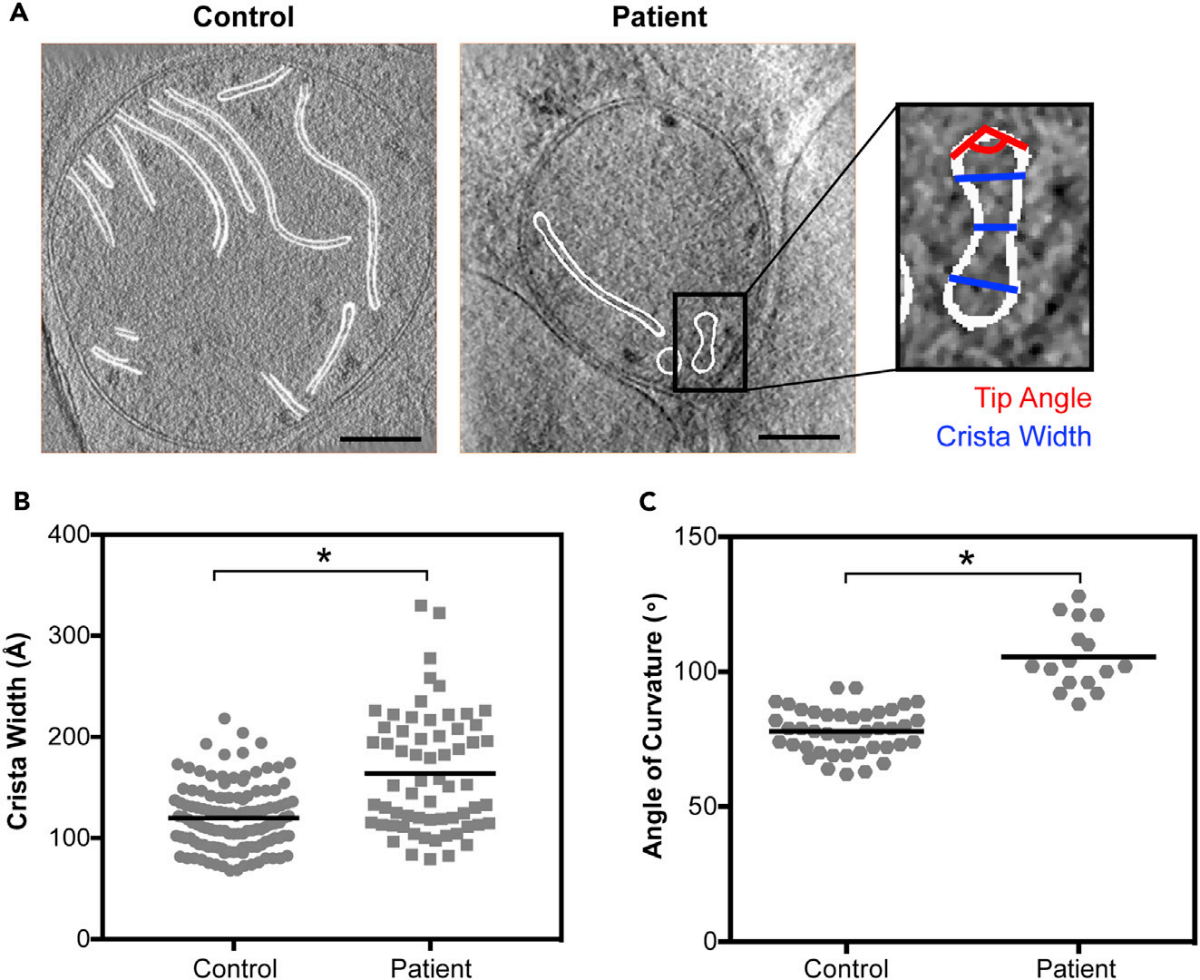
Two-Dimensional Measurements of Patient Crista Architecture Reveal Increased Width and Blunted Apex Curvature, Related to Figures S2–S4 and Videos S1, S2, S3, and S4 (A) Representative two-dimensional slices through cryoelectron tomograms, outlining cristae (white) for two-dimensional measurement of crista lumen width at base, middle, and tip (inset; blue) and of crista apex angle (inset; red); scale bars represent 200 nm. (B) Quantitation of crista lumen width in Å for control (n = 41 cristae) and patient (n = 24 cristae); ^∗^ indicates p < 0.0001. (C) Quantitation of crista apex angle for control (n = 37 cristae) and patient (n = 12 cristae). Black bars denote averages. For all graphs, significance was assessed by the Mann-Whitney test at 95% confidence. Data were collected from at least three independent cells grown on three independent grids for both patient and control; * indicates p < 0.0001. * indicates statistical significance.

Video S1. Aligned Cryo-ET Tilt Series of an *In Situ* Mitochondrion from a Healthy Human Control Subject, Related to Figure 2 A representative example of a 3×-binned tilt series of an *in situ* mitochondrion visualized by cryo-ET from skin fibroblasts of a healthy human control subject.

Video S2. Aligned Cryo-ET Tilt Series of an *In Situ* Patient Mitochondrion, Related to Figure 2 A representative example of a 3×-binned tilt series of an *in situ* mitochondrion from skin fibroblasts of the patient.

Video S3. Three-Dimensional Model of an *In Situ* Mitochondrion from a Healthy Human Control Subject, Related to Figure 2 and Figure 3 Video of a representative three-dimensional model of an *in situ* mitochondrion from skin fibroblasts of a healthy human control subject. The mitochondrion was reconstructed by manual segmentation of a 3×-binned tomogram

Video S4. Three-Dimensional Model of an *In Situ* Patient Mitochondrion, Related to Figure 2 and Figure 3 Video of a representative three-dimensional model of an *in situ* mitochondrion from skin fibroblasts of the patient. The mitochondrion was reconstructed by manual segmentation of a 3×-binned tomogram.

We further characterized the patient and control mitochondria using a series of standardized mitochondrial measurements (Figure S3). No difference was observed in mitochondrial diameter (490 ± 40 nm in patient versus 580 ± 60 nm in control; p > 0.05; Figure S4A), cross-sectional area (1.63 × 10^5^ ± 0.41 × 10^5^ nm^2^ in patient versus 1.59 × 10^5^ ± 0.42 × 10^5^ nm^2^ in control; p > 0.05; Figure S4B), or MIM-MOM distance (129 ± 18 Å in patient versus 138 ± 14 Å in the control; p > 0.05; Figure S4C), when comparing patient and control mitochondria.

As ATP synthase dimers have been implicated in the formation and maintenance of cristae (Davies et al., 2012), we analyzed crista architecture. Inspection of mitochondria from both the control (Figures S2A–S2J) and patient (Figures S2K–S2T) revealed a predominantly lamellar topology with tubular crista junctions (Figure S2), consistent with previous descriptions (Mannella et al., 2013). However, the patient's mitochondria exhibited profound ultrastructural defects compared with those from the control, including bloated, balloon-like cristae (Figures 1B, 1D, and S2K–S2T).

To characterize this abnormal morphology of patient mitochondria, we measured average crista lumen width and crista apex angle (i.e., crista tip curvature) (Figures 2A and S3). We found that crista lumen width was significantly increased in the patient (164 ± 59 Å in patient versus 120 ± 32 Å in healthy control; p < 0.0001), with a wider variation in values compared with control (∼250 Å range in patient versus 150 Å range in control) (Figure 2B). In addition, previous studies of ATP synthase have localized dimers to crista tips, pointing to an important role in establishing the apex angle of curvature (Davies et al., 2011). We therefore measured the apex angle in all cristae visible in these micrographs and found that patient cristae were significantly blunted when compared with control cristae (105° ± 12° angle of curvature in patient versus 78° ± 8° in control; p < 0.0001; Figure 2C). Of note, crista junction width was conserved between patient and control (261 ± 45 Å in patient versus 290 ± 77 Å in control; p > 0.05; Figure S4D), and values were consistent with those previously reported (250–300 Å) (Mannella et al., 2013, Quintana-Cabrera et al., 2018).

### Three-Dimensional Analyses Reveal Diminished Total Cristae Volume in Patient Mitochondria

**Figure 3:**
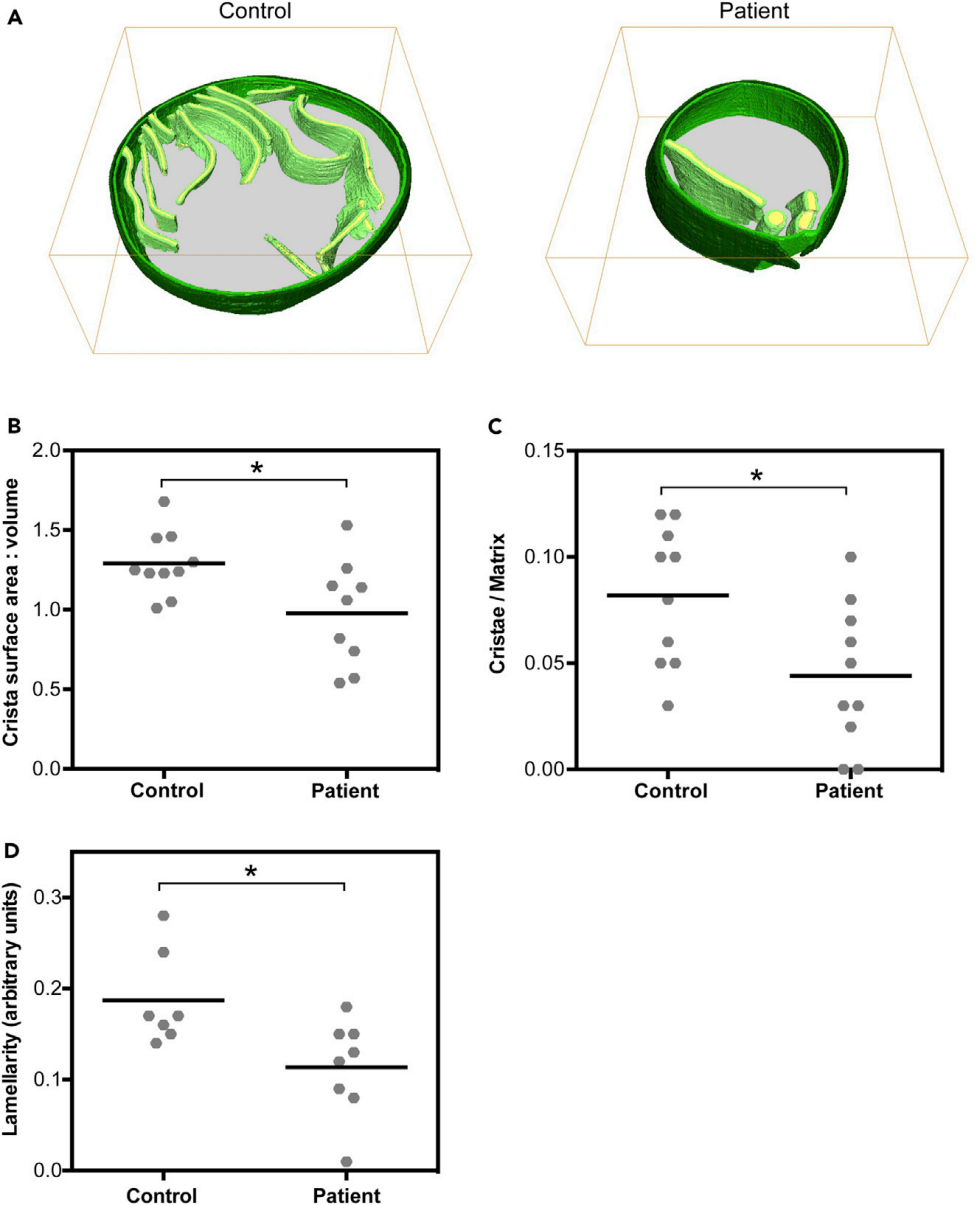
Three-Dimensional Analysis of Reconstructed Patient Mitochondria Reveals Abnormal Morphology and Distribution of Cristae, Related to Videos S3 and S4 (A) Representative three-dimensional reconstruction of *in situ* mitochondria in tomograms from control and patient fibroblasts. The latter demonstrate abnormally bloated cristae. (B) Quantitation of the surface area-to-volume ratio of total cristae membrane (light green) surface area to intracristal space (yellow) volume; * indicates p = 0.03. (C) Quantitation of crista content, expressed as total cristae volume (membrane [light green in (A)] + intracristal space [yellow in (A)]) per matrix volume [gray in (A)]; * indicates p = 0.02. (D) Quantitation of crista lamellarity, expressed as the ratio of surface area of the crista membrane to the crista lumen volume. In all calculations, we examined n = 10 mitochondria for control and n = 10 for patient; * indicates p = 0.02. For all graphs, significance was tested by the Mann-Whitney test at 95% confidence. Data were collected from at least three independent cells grown on three independent grids for both patient and control. * indicates statistical significance.

Visual inspection revealed a dearth of cristae within patient mitochondria compared with those of the healthy human control (Figures 3A and S2; Videos S3 and S4), which we further characterized using three-dimensional analyses. Visualization of mitochondrial cristae can depend greatly on biological factors (e.g., heterogeneous distribution of cristae throughout a mitochondrion) as well as methodological factors, including the angle at which the micrograph was recorded (e.g., difficulty in resolving membranes perpendicular to the axis of the electron beam in the reconstructed tomogram [Palmer and Löwe, 2014]). Therefore, quantitation of the total volume of all cristae, relative to total mitochondrial volume, was performed using three-dimensional measurements within reconstructed tomograms. Crista surface area relative to total volume occupied by all crista components was significantly decreased in patient mitochondria compared with the control (0.98 ± 0.33 au in patient versus 1.29 ± 0.20 au in control; p = 0.03) (Figure 3B), providing further evidence that absence of ATP synthase dimerization has profound effects on crista architecture. Importantly, we also found a significant decrease in total cristae volume in patient versus control mitochondria (0.04 ± 0.03 au in patient versus 0.08 ± 0.03 au in control; p = 0.02) (Figure 3C). Lastly, we measured the crista lamellarity (i.e., crista shape factor; defined as the surface area of the crista membrane/crista lumen volume) (Cserép et al., 2018) in patient and control mitochondria. Consistent with our above-mentioned findings, crista lamellarity within patient mitochondria was significantly diminished compared with control mitochondria (0.11 ± 0.05 in patient versus 0.19 ± 0.05 in control; p = 0.02) (Figure 3D).

## Discussion

Analysis of mitochondrial structure has long been integral to the understanding and diagnosis of human OxPhos diseases like LS. Here, we apply *in situ* cryo-ET to a clinical case-control study of a human mitochondrial disease, enabling the three-dimensional study of near-native patient and healthy human control samples without the significant preparation artifacts that have traditionally hindered such studies in the past (Davies and Daum, 2013, Förster and Koster, 2017). In examining healthy human control mitochondria, we have now characterized the dominant crista architecture in primary human cells under near-native conditions. These organelles are composed of tubular and lamellar cristae occupying a minority of mitochondrial volume (Lea et al., 1994, Quintana-Cabrera et al., 2018). Notably, despite use of a direct electron detection camera, we remained limited in our ability to accurately resolve the individual ATP synthase structures within our *in situ* dataset. We attributed this in part to the imaging parameters (e.g., defocus) necessary for sufficient contrast to clearly identify mitochondrial morphology and ultrastructure within the relatively thick intracellular regions examined. Therefore, rather than the angle inferred by direct visualization of ATP synthases (in dimeric or monomeric forms), our angle measurements reflect the curvature of the membrane itself at the crista tips. However, given that our value is similar to that seen in *Polytomella* (attributable to a 70° dimer angle), we believe it is likely that the ATP synthase dimers themselves exhibit an angle within the previously reported range (50° in *Euglena gracilis* [Muhleip et al., 2017], 65° in *Trypanosoma brucei* [Muhleip et al., 2017], 86° in *Saccharomyces cerevisiae* [Davies et al., 2011], and ∼86° in *Bos taurus* [Jiko et al., 2015]).

Our *in situ* cryo-ET analysis of the patient's mitochondria provides strong evidence that maintenance of crista architecture depends on ATP synthase dimerization and its absence produces profound ultrastructural defects, particularly in the setting of human OxPhos disease. The patient's bloated cristae reflect both an increased crista lumen width and an increased tip angle. This blunted curvature at the crista tips (105 ± 12° in the patient compared with 78 ± 8° in the control) is consistent with a role for dimers in establishing high crista curvature, confirming earlier work in yeast (Davies et al., 2012). The decrease in total cristae volume in the patient mitochondria also strongly supports the importance of ATP synthase dimerization in forming cristae (Paumard et al., 2002). Because the patient's mutation results in “leaky” expression of DAPIT, it is also likely that low amounts of residual protein are responsible for generating the few cristae that are observed. Nevertheless, as crista junction widths were unchanged, it also remains possible that ATP synthase dimers do not play a major role in regulating the formation of this structure. Indeed, crista architecture can be influenced by extra-mitochondrial factors, including the organelle's subcellular localization and its interaction with other intracellular structures. However, our datasets of both patient and control mitochondria included a comparable distribution of spheroid and tubular/elongated mitochondria that maintained a predominantly lamellar crista topology connected to the inner boundary membrane by tubular crista junctions, in accordance with previous observations in fibroblasts (Lea et al., 1994, Quintana-Cabrera et al., 2018). Because all mitochondria were imaged in comparable peripheral regions of fibroblasts where the mitochondrial network was indistinguishable between patient and control (as determined by fluorescence microscopy), we consider it unlikely that the clinical phenotype associated with the patient's LS can be ascribed to differences in mitochondrial morphology influenced by other subcellular factors, including those in the mitochondrial matrix.

**Figure 4:**
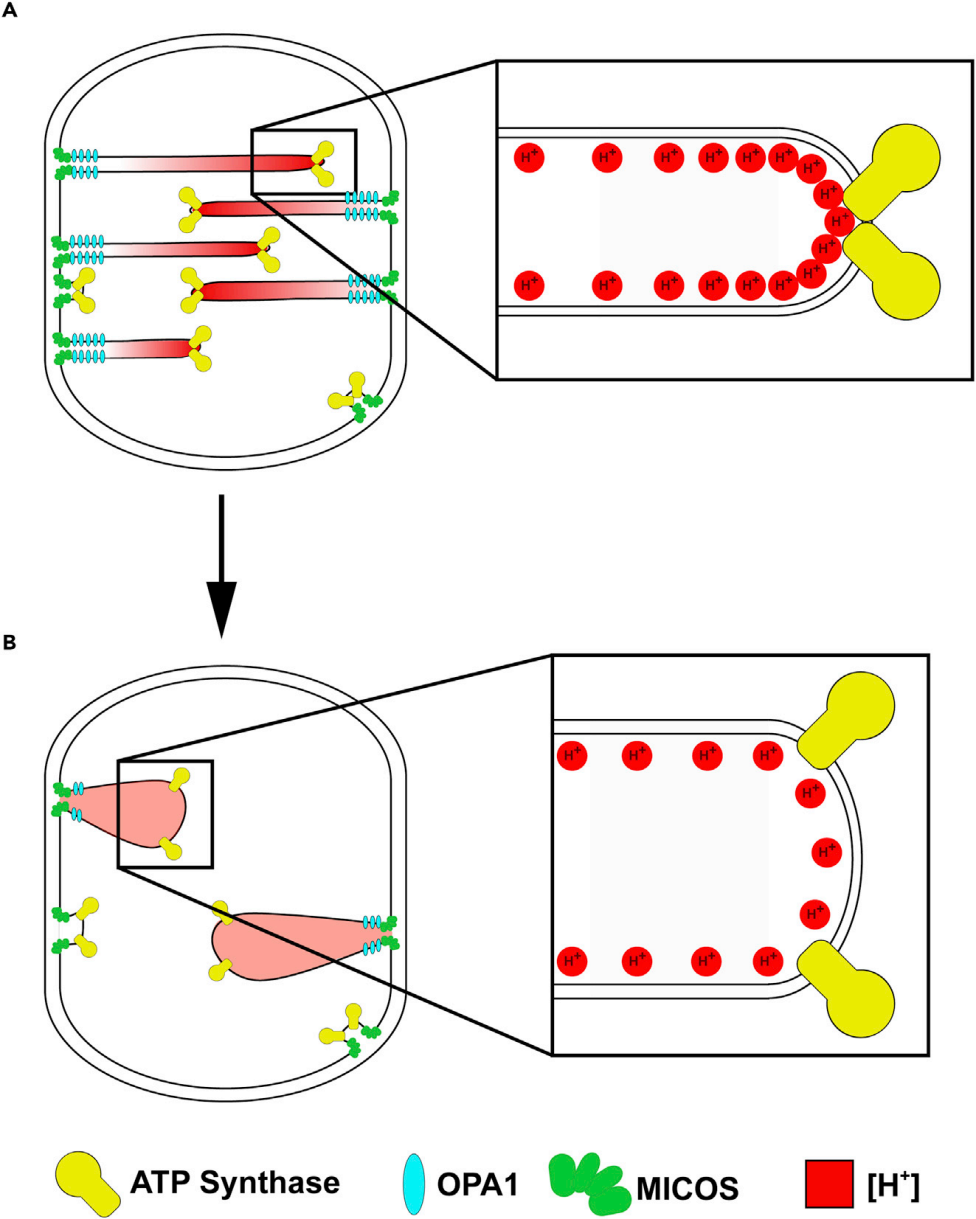
Model of Patient Pathogenic Mutation (A and B) Model representing crista architecture of mitochondria in fibroblasts from the (A) healthy control and (B) patient. In mitochondria from the healthy human control, crista structure is regulated by several factors including MICOS (green), OPA1 (blue), and ATP synthase (yellow). Protons (red) are concentrated at crista tips, as shown in the inset. By contrast, the patient's mitochondria demonstrate fewer cristae, as well as an increased crista tip angle of curvature and increased crista lumen width. Crista junction width is unaffected. The pathogenic changes to the crista architecture may impair the mitochondria's capacity to concentrate protons efficiently at crista tips, resulting in a lower ΔpH and decreased protonmotive force necessary to generate energy. Model adapted from Strauss et al. (2008) and Cogliati et al. (2016).

Importantly, the work described here provides support for a unified pathogenic mechanism in LS by linking the insufficient ATP production caused by an ATP synthase dimerization defect to structural defects in crista architecture. We have recently come to appreciate the higher order organization of respiratory complexes, which are concentrated within the cristae (Gilkerson et al., 2003) and arranged into multicomplex associations of predefined composition, including ATP synthase dimers (Allen et al., 1989, Althoff et al., 2011, Davies et al., 2011, Gu et al., 2016, Schagger and Pfeiffer, 2000). Significantly, we recently showed that the patient's mutation affected ATP synthesis *in vivo* by disrupting this ATP synthase dimerization, rather than by interfering with ATP synthesis catalytic activity per se (Barca et al., 2018). Now, we implicate this disease-causing mutation in a disruption of crista topology, which provides putative mechanisms by which the dimerization defect might impair ATP synthesis (Figure 4). For example, highly curved cristae formed by rows of ATP synthase dimers are thought to concentrate respiratory chain-derived protons, thereby maximizing the proton gradient (ΔpH) powering ATP synthesis (Strauss et al., 2008). By contrast, the patient's markedly abnormal cristae might concentrate protons less efficiently at crista tips, resulting in a lower ΔpH and therefore a decreased protonmotive force. Also, the dimerization impairment might be impairing crista formation, causing the patient's comparatively cristae-deficient mitochondria's lower respiratory capacity (Nielsen et al., 2017). It has been shown that ATP synthase interacts with components of the mitochondrial contact sites and cristae-organizing system (MICOS) machinery responsible for generating cristae (Rampelt et al., 2017), and our results open the door for future studies to investigate the role of DAPIT in this interaction. Overall, our findings highlight the role of mitochondrial ultrastructure in bioenergetic output *in vivo*.

In the midst of the current cryo-EM revolution, maximum resolution continues to improve dramatically, although to date much of the implementation of this technique has focused on highly purified samples. Our application of *in situ* cryo-ET to human mitochondrial disease suggests the promise of a novel precision medicine-focused approach, where the study of pathogenic mutations in readily accessible and minimally invasive patient samples may reveal important, yet subtle changes visible only under near-native conditions.

## Methods

All methods can be found in the accompanying Transparent Methods supplemental file.

## Supplemental Information

Document S1. Transparent Methods and Figures S1–S4
