# Supplementary material for: Three-Dimensional Analysis of Mitochondrial Crista Ultrastructure in a Patient with Leigh Syndrome by In Situ Cryoelectron Tomography
Source: iScience. 2018 Jul 20;6:83–91. doi: 10.1016/j.isci.2018.07.014 (PMC6137323; doi:10.1016/j.isci.2018.07.014)
Supplement: Document S1. Transparent Methods and Figures S1–S4 [file mmc1.pdf]

ISCI, Volume *6*

Supplemental Information

Three-Dimensional Analysis of Mitochondrial Crista Ultrastructure in a Patient with Leigh Syndrome by *In Situ* Cryoelectron Tomography

Stephanie E. Siegmund, Robert Grassucci, Stephen D. Carter, Emanuele Barca, Zachary J. Farino, Martí Juanola-Falgarona, Peijun Zhang, Kurenai Tanji, Michio Hirano, Eric A. Schon, Joachim Frank, and Zachary Freyberg

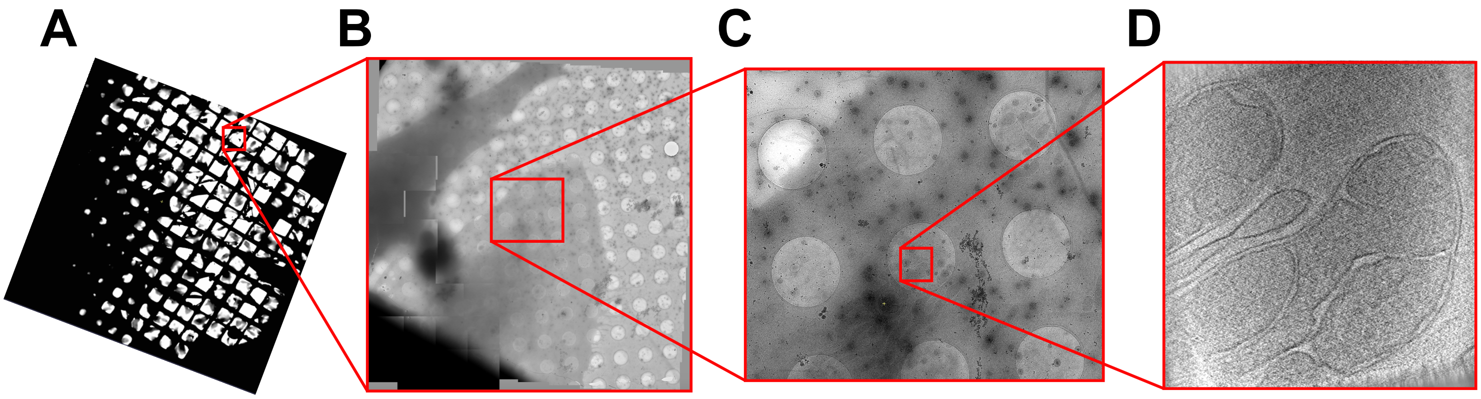

Figure S1. Imaging of mitochondria in fibroblasts grown on electron microscopy grids, Related to Figure 1. (**A**) Patient and control skin fibroblasts were grown directly on London finder grids. (**B**) Cells with thin, cryo-EM accessible regions (<500 nm) were identified. Within these regions, mitochondria were identified (**C**) and all mitochondria located over holes were imaged as in (**D**).

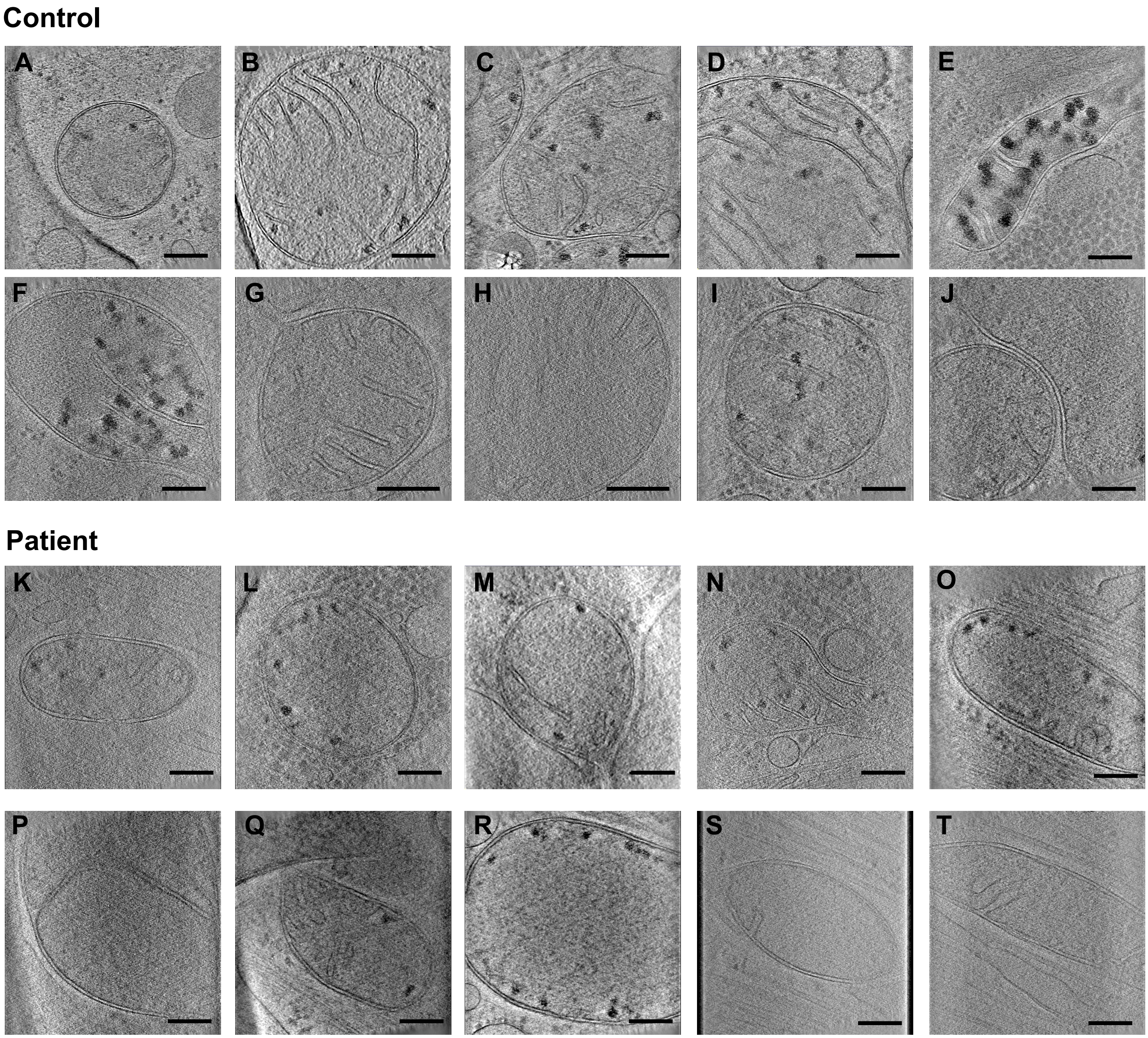

Figure S2. Representative images of the entire dataset of *in situ* mitochondria from control and patient fibroblasts, Related to Figure 1 and Figure 2. A slice taken from the center of each control (**Panels A-J**) and patient (**Panels K-T**) tomogram is shown, demonstrating the entire dataset of peripheral spherical and tubular/elongated mitochondria collected. Differences in overall crista architecture (crista paucity, bloated and blunted crista) were seen in patient mitochondria compared with those from the control. Scale bars = 200 nm.

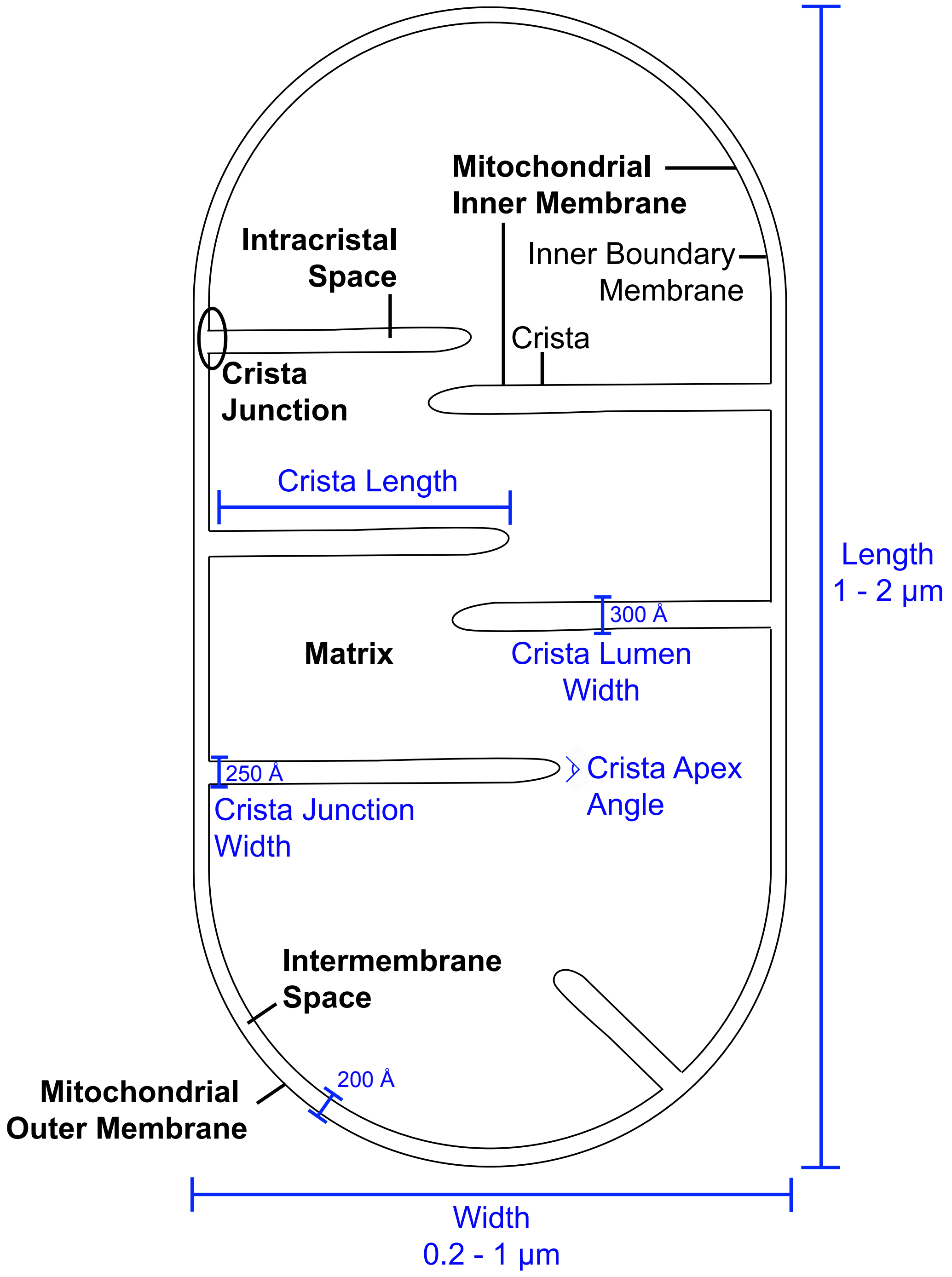

Figure S3. Diagram of standardized mitochondrial measurements, Related to Figure 2. Mitochondrial components (black) and measurement parameters for characterizing crista architecture (blue) are shown, along with respective previously described values.

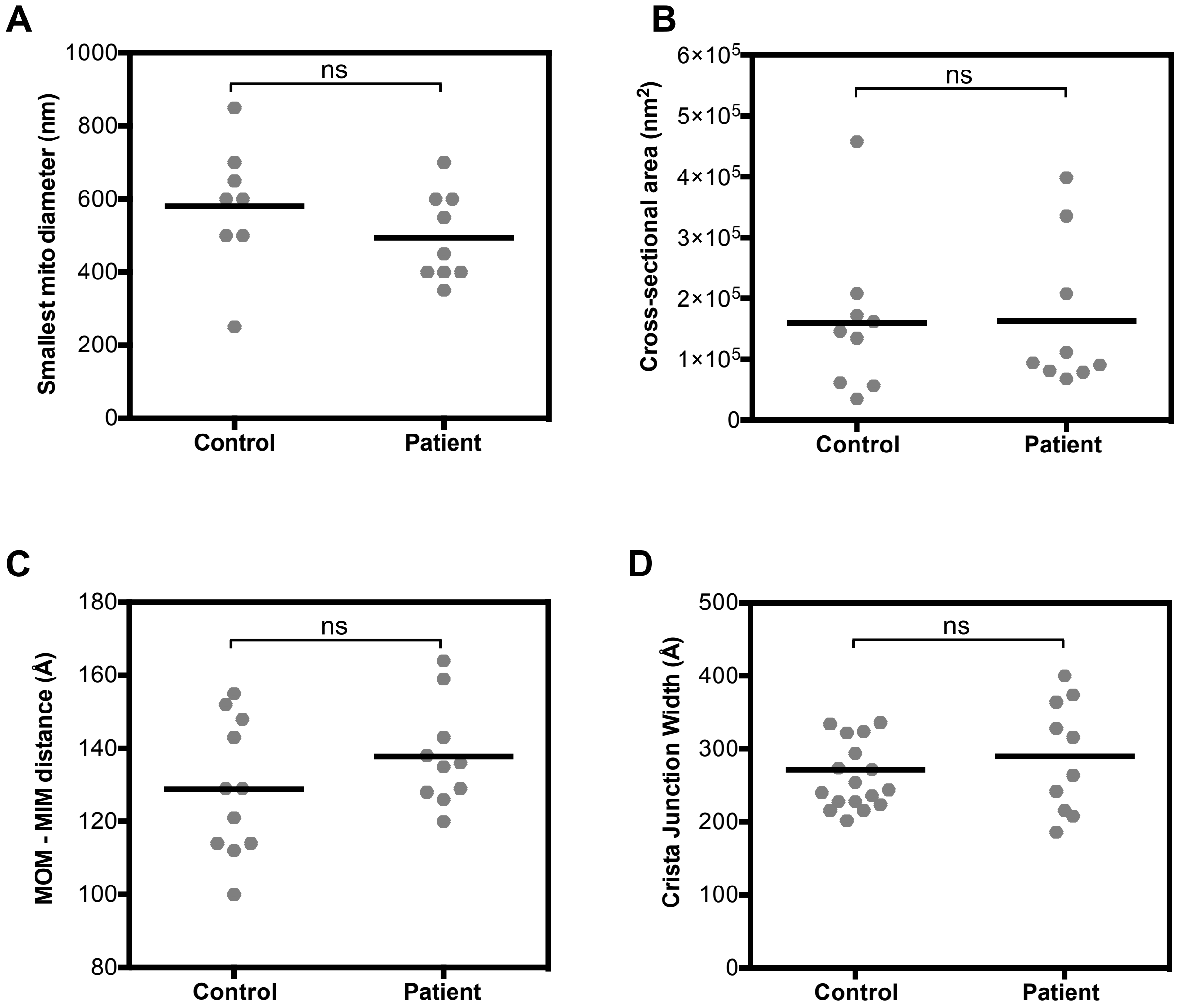

Figure S4. Mitochondrial size, MOM-MIM distance and crista junction width are unaffected by the USMG5 mutation in the patient, Related to Figure 2. (**A**) Quantitation of smallest mitochondrial diameter (defined as the diameter of spherical or width of tubular/ elongated mitochondria) in nm for control (n=8) and patient (n=9) mitochondria. Of note, two control and one patient mitochondria extended beyond the tomogram and size could not be determined. (**B**) Quantitation of mitochondrial cross-sectional area in nm^2 for control (n=8) and patient (n=9) mitochondria. (**C**) Quantitation of the average MOM-to-MIM distance for control (n = 10 mitochondria) and patient (n = 10 mitochondria); each point represents the average of four measurements (taken at four equally spaced points, corresponding approximately to 0°, 90°, 180°, and 270° positions). (**D**) Quantitation in Å of the cristae junction width for control (n = 18 crista junctions) and patient (n = 10 crista junctions). Significance tested by Welch's t-test at 95% confidence.

TRANSPARENT METHODS

Cell Culture

We obtained skin fibroblasts from the patient expressing a homozygous *USMG5* mutation (C.87+1G>C, 1 basepair after the end of Exon 3), and from a healthy human subject. All subjects provided written consent and all studies were performed in accordance with a Columbia University Medical Center (CUMC) IRB-approved protocol (#AAAJ8651). Skin biopsy to obtain the fibroblast cells was only performed following informed consent. The respective cells were cultured as previously described (Barca et al., 2018). Briefly, fibroblasts were cultured in Dulbecco's minimal essential media (DMEM) (Thermo Fisher, Waltham, MA, USA) supplemented with 15% fetal bovine serum (FBS) (Sigma-Aldrich, St. Louis, MO, USA), 1% vitamin solution and 1% antibiotic-antimycotic (Thermo Fisher). All experiments were conducted on cells cultured for <15 passages.

Confocal Fluorescence Microscopy

For live fluorescence light microscopy, fibroblasts were plated 24 h prior to imaging in a 35-mm uncoated glass-bottom culture dish (MatTek Corp., Ashland, MA, USA) at a density of ~30% coverage. Cells were stained with MitoTracker Green FM (Thermo Fisher) and imaged live on a *Leica SP5* II laser scanning *confocal microscope* (Leica Camera AG, Wetzlar, Germany) using a 63x objective at the Confocal and Specialized Microscopy Shared Resource of the Herbert Irving Comprehensive Cancer Center at Columbia University.

Cryo-electron tomography

Sample preparation. Cells were plated for 24-48 h on fibronectin-coated Quantifoil R 2/2 holey carbon Au 200-mesh or London Finder grids (Electron Microscopy Sciences, Hatfield, PA, USA) to a density of ~30% coverage and grown under normal culture conditions. Immediately prior to vitrification, BSA-coated 20-nm gold beads (Sigma-Aldrich) were applied to grids as fiducial markers, and samples were then plunge-frozen in liquid ethane using a Vitrobot Mark IV (FEI Thermo Fisher, Hillsboro, OR, USA) with a blot force of 1 and a blot time of 8 sec using Whatman #1 filter paper at 100% humidity and 22°C.

Cryo-electron tomography. Imaging was conducted on a Tecnai F30 Polara (FEI Thermo Fisher) microscope operating at 300 kV, with a Gatan K2 Summit direct electron detection (DED) camera (Gatan, Pleasanton, CA, USA) in super-resolution mode. Images were collected using SerialEM (version 3; Boulder, CO, USA) (Mastronarde, 2005) and then binned down 2x to achieve a pixel size of 2.6 Å. Tilt series were collected using a bidirectional tilt scheme from 0°±60° with 1.5° increments and a defocus range of -5.0 to -6.0 µm. The cumulative total electron dose of each tilt series was 100-180 $e^-/Å^2$. Tilt series were subsequently aligned and reconstructed using the IMOD software package (version 4.9; Boulder, CO, USA) (Kremer et al., 1996). All identified mitochondria were imaged (11 patient and 18 gender-matched control mitochondria in total), and analysis was limited for both patient and control samples to the tilt series with highest signal-to-noise ratio (SNR) as determined by cross-correlation-based alignment (n=10 for each group) using IMOD software.

Representative data were collected from at least three independent cells grown on three independent grids for both patient and control samples.

Ultrastructural characterization

Ultrastructural characterization of mitochondria was performed using the Amira software package (Thermo Fisher FEI). All analyses were blinded to sample identity. Measurements were made according to the diagram shown in **Fig. S3**.

Two-dimensional analyses. Two-dimensional analysis and quantitation were performed for each mitochondrion in a two-dimensional slice taken from the middle of the corresponding 3x-binned tomograms (8 pixels averaged together). All two-dimensional quantitative measures were calculated using the Amira software package. Mitochondrial size was measured using two parameters: (1) along the shortest dimension, and (2) cross-sectional area. The crista apex angle was measured by placing a vertex at the extreme tip of a crista and connecting this to each crista membrane at the points where the membranes became most closely parallel. Crista width was measured by placing points directly opposite each other at the outer boundary of each of the two membranes comprising a crista and measuring the distance spanning the outer margins. For crista continuous with the inner boundary membrane (IBM), measurements were made near but not at the crista junction, which was measured separately. The distance between the MIM and MOM was measured for each mitochondrion by placing directly-apposed points on the MIM and MOM, as close as possible to the approximate 0°, 90°, 180°, and 270° positions.

Three-dimensional analyses. Tomograms were aligned using the IMOD software package (Mastronarde and Held, 2017) (**Videos S1 and S2**), utilizing fiducial markers (whenever at least 5 fiducials were visible throughout a tilt series) or fiducial-free cross-correlation. Three-dimensional volume reconstruction of 3x-binned tilt series was then conducted via a simultaneous iterative reconstruction technique (SIRT), using Tomo3D (Agulleiro and Fernandez, 2011). Volumes were subsequently rotated in IMOD to retain chirality while re-assigning the z-axis to that of the electron beam.

Manual segmentation of mitochondrial membranes, as well as subsequent three-dimensional modeling, quantitation, and video generation, were performed on 3x-binned tomograms using the Amira software package. To quantitate total cristae volume per mitochondrion, we determined the upper and lower boundary slices in the tomogram in which the entire MOM and adjacent IBM could faithfully be traced. Measurement was limited to the volume within these boundaries to ensure that total mitochondrial volume could be calculated and compared with crista volume; while all mitochondria were intact within their native environment, limitations inherent to TEM result in the upper and lower margins of each organelle from being visualized and this is the reason for the restriction of our measurements to segments where the entire boundary of the organelle could be determined. We then measured (1) the total volume within the IBM, including matrix and cristae (“mitochondrial volume”); and (2) the total volume occupied by all crista components, including crista membrane and intracristal space (“crista volume”). Cristae volume was divided by mitochondrial volume to calculate crista volume per mitochondrion. To quantitate crista surface-to-volume ratio, we subdivided the total

crista volume into cristal membrane and intracristal space. To measure the crista lamellarity (i.e. crista shape factor), we divided the surface area of the crista membrane by the crista lumen volume as described previously (Cserep et al., 2018).

Statistics

Statistics was performed using GraphPad (version 6.0e for Macintosh; GraphPad Software, La Jolla, CA, USA). All datasets were tested for normality using the D'Agostino and Pearson omnibus normality test, with $\alpha<0.05$ constituting a normal distribution. The crista width measurement dataset was found to have a non-normal distribution and was therefore analyzed for significance using the non-parametric Mann-Whitney test, with $p<0.05$ defining a significant difference; crista lamellarity was similarly analyzed using the Mann-Whitney test. All other measurement datasets followed a Gaussian-like distribution and were analyzed using the parametric Welch's t-test, with significance defined as $p<0.05$.

Supplemental References

Agulleiro, J.I., and Fernandez, J.J. (2011). Fast tomographic reconstruction on multicore computers. Bioinformatics (Oxford, England) *27*, 582-583.

Barca, E., Ganetzky, R.D., Potluri, P., Juanola-Falgarona, M., Gai, X., Li, D., Jalas, C., Hirsch, Y., Emmanuele, V., Tadesse, S.*, et al.* (2018). USMG5 Ashkenazi Jewish founder mutation impairs mitochondrial complex V dimerization and ATP synthesis. Hum Mol Genet. Jun 18. doi: 10.1093/hmg/ddy231.

Cserep, C., Posfai, B., Schwarcz, A.D., and Denes, A. (2018). Mitochondrial Ultrastructure Is Coupled to Synaptic Performance at Axonal Release Sites. eNeuro *5*.

Kremer, J.R., Mastronarde, D.N., and McIntosh, J.R. (1996). Computer visualization of three-dimensional image data using IMOD. J Struct Biol *116*, 71-76.

Mastronarde, D.N. (2005). Automated electron microscope tomography using robust prediction of specimen movements. Journal of structural biology *152*, 36-51.

Mastronarde, D.N., and Held, S.R. (2017). Automated tilt series alignment and tomographic reconstruction in IMOD. Journal of structural biology *197*, 102-113.
